# Sudomotor Dysfunction of Feet Is Associated with Cardiac Autonomic Neuropathy in Patients with Type 2 Diabetes: A Cross-Sectional Study

**DOI:** 10.3390/medicina61101848

**Published:** 2025-10-15

**Authors:** Alexandra Gogan, Sandra Lazar, Ovidiu Potre, Vlad-Florian Avram, Andreea Herascu, Minodora Andor, Florina Caruntu, Bogdan Timar

**Affiliations:** 1 Doctoral School of Medicine, “Victor Babes” University of Medicine and Pharmacy, 300041 Timisoara, Romania; alexandra.gogan@umft.ro (A.G.); andreea.herascu@umft.ro (A.H.); 2 First Department of Internal Medicine, Medical Semiology II, “Victor Babes” University of Medicine and Pharmacy, 300041 Timisoara, Romania; andor.minodora@umft.ro (M.A.); caruntu.florina@umft.ro (F.C.); 3 Cardiology Clinic, Institute of Cardiovascular Disease, 300310 Timisoara, Romania; 4 First Department of Internal Medicine, Hematology, “Victor Babes” University of Medicine and Pharmacy, 300041 Timisoara, Romania; potre.ovidiu@umft.ro; 5 Department of Hematology, Emergency Municipal Hospital, 300254 Timisoara, Romania; 6 Centre for Molecular Research in Nephrology and Vascular Disease, “Victor Babes” University of Medicine and Pharmacy, 300041 Timisoara, Romania; avram.vlad@umft.ro (V.-F.A.); bogdan.timar@umft.ro (B.T.); 7 Multidisciplinary Research Centre for Malignant Hematological Disease (CCMHM), “Victor Babes” University of Medicine and Pharmacy, 300041 Timisoara, Romania; 8 Department of Diabetes, “Pius Brinzeu” Emergency Hospital, 300736 Timisoara, Romania; 9 Second Department of Internal Medicine, “Victor Babes” University of Medicine and Pharmacy, 300041 Timisoara, Romania; 10 Multidisciplinary Heart Research Center, “Victor Babes” University of Medicine and Pharmacy, 300041 Timisoara, Romania; 11 Cardiology Clinic of Timisoara Municipal Clinical Emergency Hospital, 300040 Timisoara, Romania

**Keywords:** cardiac autonomic neuropathy, sudomotor dysfunction, SUDOSCAN, type 2 diabetes mellitus

## Abstract

*Background and Objectives*: Cardiac autonomic neuropathy (CAN) is a common but also underdiagnosed complication of diabetes mellitus (DM), associated with high cardiovascular risk and mortality. Sudomotor dysfunction can serve as an early indicator of autonomic dysfunction. This study evaluated the association between sudomotor dysfunction and the severity of CAN in patients with type 2 diabetes (T2D). *Materials and Methods*: In this cross-sectional study, 109 patients with T2D were evaluated for diabetic peripheral neuropathy, cardiovascular autonomic dysfunction, and sudomotor dysfunction. Additionally, clinical and biochemical data were collected from patients’ medical records. *Results*: Sudomotor dysfunction (SUDO+) was present in 59.6% of patients. The presence of SUDO+ was associated with a higher age, longer duration of diabetes, lower eGFR (estimated glomerular filtration rate) values, and more severe signs of peripheral neuropathy. SUDO+ patients showed significantly greater orthostatic systolic and diastolic BP (blood pressure) changes, lower RR interval ratios, and lower feet ESC (electrochemical skin conductance) values. ROC (receiver operating characteristic) analysis for feet ESC in identifying pathological RR ratio showed an AUC of 0.689 (95% CI: 0.593–0.774, *p* = 0.0022), with a sensitivity of 46.7% and a specificity of 94.7% at a cutoff of ≤68 µS. For orthostatic hypotension and QTc prolongation, the ESC values had limited discriminative power. Chi-squared analysis showed a significant association between feet sudomotor impairment and pathological RR ratio (χ^2^ = 6.521, *p* = 0.0107). *Conclusions*: Sudomotor dysfunction is associated with indicators of CAN. SUDOSCAN can be used as a complementary tool for early CAN detection in clinical practice.

## 1. Introduction

Autonomic and peripheral neuropathy are the most prevalent chronic complications of diabetes mellitus (DM) [1]. Despite having a substantial influence on the survival and quality of life of diabetics, diabetic autonomic neuropathy (DAN) is one of the least known and understood consequences of diabetes [2]. DAN may negatively impact the cardiovascular, gastrointestinal, genitourinary, and neurovascular systems.

One of the earliest manifestations of DAN is thought to be sudomotor dysfunction. Sudomotor function is predominantly regulated by tiny, unmyelinated cholinergic sympathetic C-fibers that control cardiovascular autonomic function and may be affected earlier in patients experiencing diabetic neuropathy (DN). In order to detect autonomic neuropathy in diabetes patients early on, the American Diabetes Association (ADA) has incorporated sudomotor function [3]. Because DM is the most common metabolic disease in the world and a major cause of cardiovascular risk, evaluating sudomotor function may be useful to identify those who are more likely to suffer neuropathic and cardiovascular implications [4].

Clinical tests for DN, as is well known, mostly include examining various peripheral nerves, typically the big type A alpha and beta myelinated nerve fibers. However, due to the lack of adequate assessment techniques, the sympathetic nervous system’s thin, unmyelinated type C fibers are typically disregarded. As demonstrated in patients with early diabetes using various techniques, such as skin biopsies, sweat gland activity regulated by sympathetic C fibers may be impacted in the early pathological development of diabetes or prediabetes [5,6].

The SUDOSCAN device has been developed to address these diagnostic challenges by providing a quick, simple, and non-invasive method of measuring electrochemical skin conductance (ESC). The SUDOSCAN gadget has been introduced as an innovative approach for the early identification of DAN, specifically for the detection of cardiac autonomic neuropathy, by evaluating sudomotor function [7,8,9].

The objective of the study was based on the fact that sudomotor dysfunction, shown by low ESC values, may be associated with CAN in patients diagnosed with T2D. The study aimed to explore the relationship between ESC values obtained using SUDOSCAN, with heart rate response to deep breathing, orthostatic blood pressure response, and QT interval, which are clinical markers of CAN. In addition, the study explored how sudomotor dysfunction varied with clinical and metabolic markers, such as duration of diabetes, body mass index (BMI), hemoglobin A1c (HbA1c), renal function, standard tests for peripheral neuropathy, and presence of microvascular complications or major cardiovascular events. Another objective of the study was to evaluate the diagnostic performance of ESC values in the detection of cardiac autonomic dysfunction by analyzing the sensitivity and optimal cutoff points.

Afterwards, this study aims to determine whether SUDOSCAN provides clinically relevant information for the detection of CAN, in addition to the usual diagnostic tools.

## 2. Materials and Methods

### 2.1. Study Design and Patients

This cross-sectional, observational study was conducted at the Diabetes, Nutrition, and Metabolic Diseases Department of the “Pius Brînzeu” Emergency Clinical County Hospital in Timișoara, Romania, between September and December 2024.

During normal hospital admissions, 109 adult patients with confirmed T2DM were consecutively enrolled. The diabetes department admitted all patients for clinical assessment or treatment optimization. The following criteria were used for inclusion: (1) age 18 years or older; (2) a diagnosis of T2DM, as defined by the American Diabetes Association’s (ADA) guidelines [10]: plasma glucose levels of 126 mg/dL or higher during fasting, 200 mg/dL or HbA1C ≥ 6.5% during two hours, and 200 mg/dL during hyperglycemic symptoms or a crisis; and (3) the capacity and willingness to undergo a thorough autonomic and sudomotor evaluation. The patients with another type of diabetes, pregnant women, and those with a history of neoplasm, history of non-diabetic neurological disorders, presence of cardiac arrhythmias, pacemaker, advanced heart failure (NYHA III-IV), or limb amputation were excluded.

The primary outcome of this study was the association between sudomotor dysfunction and markers of CAN. Secondary outcomes included the relationship between sudomotor dysfunction and clinical/metabolic characteristics, as well as diagnostic performance of ESC through ROC analysis.

The study was conducted in accordance with the principles of the Helsinki Declaration. Written informed consent was signed by each patient before they were enrolled in the clinical study. The Ethics Committee of the “Victor Babeș” University of Medicine and Pharmacy in Timișoara, Romania, approved the study protocol and informed consent (No. 45 from 10 September 2024), and the study protocol was also approved by the Local Ethics Committee for Scientifical Research of “Pius Brinzeu” Emergency Hospital Timisoara (No. 410 from 27 October 2023).

### 2.2. Anthropometrical, Clinical, and Laboratory Assessments

At admission, a standardized set of clinical, laboratory tests called CARTs (cardiovascular autonomic reflex tests), were performed on all enrolled patients. Anthropometric assessments, biochemical investigations, and neurological evaluations that focused on peripheral somatic and autonomic functioning were all part of the study protocol.

#### 2.2.1. Clinical and Laboratory Investigations

Height and weight were recorded using stadiometers and body weight scales; patients were shoeless and lightly dressed. Body mass index (BMI) was calculated by dividing weight in kilograms by height in meters squared. Blood pressure (BP) and heart rate (HR) were recorded manually with a stethoscope and a calibrated sphygmomanometer, with regular verification according to the manufacturer’s recommendations. All blood pressure measurements were taken in the patient’s left hand after resting in a sitting position for a minimum of 5 min. Medical records and structured interviews were used to gather comprehensive clinical data. To determine whether patients had a personal pathologic history of major cardiovascular events, such as myocardial infarction (MI), stroke, or peripheral arterial disease (PAD), medical history was reviewed, and these data were included only if they were verified by hospital discharge or after specialist assessment. In addition, data on the duration of diabetes and current treatment such as insulin or metformin were obtained and verified by medical records. During hospitalization, patients received an ophthalmologic consultation, which included fundus examination to determine the absence or presence of diabetic neuropathy. Samples of venous blood were taken early in the morning following a minimum of eight hours of fasting and were performed in the accredited “Pius Brînzeu” Emergency County Hospital laboratory, which applies standardized methods. Glycated hemoglobin (HbA1c) and serum creatinine were the primary biochemical markers examined. The CKD-EPI formula was utilized to determine the estimated glomerular filtration rate (eGFR).

#### 2.2.2. Evaluation of Sudomotor Function

Sudomotor dysfunction was assessed using a non-invasive, FDA-approved SUDOSCAN device that measures electrochemical conductance of the skin at the palms and feet to determine sudomotor function. In this study, ESC values obtained with SUDOSCAN were analyzed and reported as indicators of sudomotor function. This method provides information about the status of small, sympathetic, unmyelinated C-fibers that innervate sweat glands and are commonly affected in early diabetic neuropathy. Patients were told to put their hands and feet on the metal electrodes as part of the procedure. Applying a low-voltage electrical stimulation creates a localized current that activates the chloride ions in the sweat ducts. The conductance, which is measured in microsiemens (µS), shows the activity of the sweat glands. The entire exam is painless, takes two to three minutes, and requires no prior preparation. The hands and feet have different ESC values. In the present study, we used the mean ESC value for the feet, with a cutoff value of less than 70 µS, and for the arms, with a cutoff value of lower than 60 µS, to determine sudomotor dysfunction in accordance with the instructions given by the device manufacturer. All measurements were made in a quiet, temperature-controlled environment, and patients were advised to avoid smoking and drinking coffee for at least four hours before the test because both can stimulate the autonomic nervous system and alter sudomotor function, potentially influencing ESC values. They were also advised to take off any shoes or hand creams that would prevent the electrodes from making appropriate contact.

#### 2.2.3. Evaluation of Cardiac Autonomic Neuropathy

Ewing tests, specifically the heart rate response to deep breathing and the blood pressure response to orthostatic transition, were used to assess CAN. The QTc interval prolongation was also use for the diagnosis of CAN. Through these tests, the sympathetic and parasympathetic aspects of autonomic function can be examined. A 12-lead electrocardiogram (ECG) was used to record the heart rate variability during deep breathing. Six cycles per minute of deep breathing were required of the patients, and the RR ratio, which is the ratio of the longest to shortest RR interval, was calculated. A ratio with a value less than 1.1 was considered abnormal. The corrected QT interval (QTc) was determined using the Bazett algorithm. In men, a value above 440 milliseconds (ms) and in women above 460 ms was considered QT prolongation. Systolic and diastolic pressures were recorded following a minute of standing for the blood pressure response to orthostatic transition test. A drop of ≥20 mmHg in systolic blood pressure (SBP) or ≥10 mmHg in diastolic blood pressure (DBP) was considered pathological.

#### 2.2.4. Evaluation of Peripheral Neuropathy

The Toronto Clinical Neuropathy Score (TCNS) and Michigan Neuropathy Screening Instrument (MNSI) were used to evaluate peripheral somatic neuropathy. The MNSI consists of two distinct evaluations: a clinical examination of the feet, specifically foot inspection, vibration sensitivity assessment with a 128-Hz tuning fork, and ankle reflex testing, conducted by a physician, and a questionnaire with 15 questions for the diagnosis of diabetic neuropathy. A maximum of eight points can be obtained for every distinctive finding. Higher scores imply more severe neuropathy. The TCNS includes reflex evaluation, symptoms scoring, and sensory testing, such as vibratory, temperature, and pinprick testing. The maximum score was 19, with higher scores indicating more severe neuropathy.

#### 2.2.5. Ankle–Brachial Pressure Index (ABPI)

PAD was screened using the ankle–brachial pressure index (ABPI), performed with MESI ABPI MD that performs automated measurements. PAD was considered to be consistent with an ABPI < 0.9.

### 2.3. Statistical Analysis

The statistical analysis was conducted using the MedCalc^®^ Statistical Software version 22.015 (MedCalc Software Ltd., Ostend, Belgium). Numerical variables with a Gaussian distribution are described by the mean ± standard deviation (SD), while those with a non-parametric distribution are described by the median and interquartile range [IQR]. Categorical variables were shown as absolute frequencies and percentages. We applied the Shapiro and Wilk method to determine whether the distribution of the numerical variables was normal. In this evaluation, a *p*-value of less than 0.05 indicates non-parametric distributions. We compared two medians using the Mann–Whitney U test to evaluate the variations between the central tendency indicators. For categorical data, the chi-square test was used, and contingency coefficients were calculated to assess the strength of associations. To assess the prediction power of sudomotor dysfunction, we conducted “receiver-operating characteristics” analyses with performance measured in terms of sensitivity and specificity. The area under the ROC curve (AUC), 95% confidence intervals (CI), and the Youden index (J) were calculated to identify the threshold value. For linear regression analyses, the model assumptions were verified and adequately satisfied. Given the relatively small sample size and the multiple statistical comparisons performed, the results should be interpreted with caution. No formal multiplicity adjustment was applied, but we focused on the primary outcome to minimize the risk of spurious associations.

The sample size calculation was performed prior to recruitment. In order to achieve a 95% confidence level and a statistical power greater than 80%, we estimated that at least 100 participants would be required. A *p*-value of 0.05 was used as the threshold for statistical significance in the present study. Therefore, we recruited 109 patients, which fulfilled the sample size requirement.

## 3. Results

In this cross-sectional study, 109 patients with a diagnosis of T2D were enrolled, of whom 51.4% (56) were male and 48.6% (53) were female. The median age of the patients was 63.6 years [56; 70], the median duration of diabetes was 12 years [6; 20], and the median HbA1c was 8.5% [7.3; 9.4], with a median body mass index of 31.3 kg/m^2^ [27.5; 34.6] and a median estimated glomerular filtration rate of 75 mL/min/1.73m^2^ [55; 88]. In addition, the majority of patients in this study were treated with insulin, 64.2% (70), and/or metformin, 65.1% (71). Diabetic retinopathy was present in 32.1% (35) of cases, history of major cardiovascular events in 16.5% (18), and peripheral arterial disease (PAD) in 32.1% (35). Sudomotor dysfunction was present in 59.6% (65) of cases and orthostatic hypotension in 36.7% (40). The characteristics of the sample studied are presented in Table 1.

Among the 109 patients, 59.6% (65) were categorized as SUDO+, indicating that sudomotor dysfunction had been significant in this study.

Patients in the group with presence of sudomotor dysfunction (SUDO+) were significantly older (65.34 vs. 61.07, *p* = 0.0098) and had lower eGFR values (65.64 vs. 79.93; *p* = 0.0085) and a longer duration of diabetes (14.43 vs. 10.45 *p* = 0.0018). Furthermore, within the same group, there were significant decreases in both systolic blood pressure (−5.98 vs. 4.23, *p* = 0.0001) and diastolic blood pressure (−3.26 vs. 3.20, *p* = 0. 0001) in response to standing, as well as a lower RR ratio (1.04 vs. 1.09, *p* = 0.0278). Scores for diabetic neuropathy were higher in patients with SUDO+, namely, MNSI (6.60 vs. 4.75, *p* < 0.0001) and Toronto (6.11 vs. 4.50, *p* < 0.0001). Table 2 shows data on the comparative analysis of sudomotor dysfunction.

In a separate analysis assessing sudomotor dysfunction of the feet (SUDO feet+) (Table 3), it was observed that patients were older (69 vs. 60, *p* = 0.0003), had diabetes for a longer duration (13 vs. 10, *p* = 0.0214), showed a decrease in RR ratio (1.04 vs. 1.11, *p* = 0.0013), and exhibited a significant decrease in both systolic (−10 vs. 5, *p* < 0.0001) and diastolic (−5 vs. 0, *p* < 0.0001) blood pressure.

Only severe peripheral neuropathy is substantially linked to sudomotor dysfunction of the arms (SUDO Arms+), as indicated by higher MNSI and Toronto scores. There were no discernible variations in autonomic or metabolic markers, as shown in Table 4.

**Figure 1:**
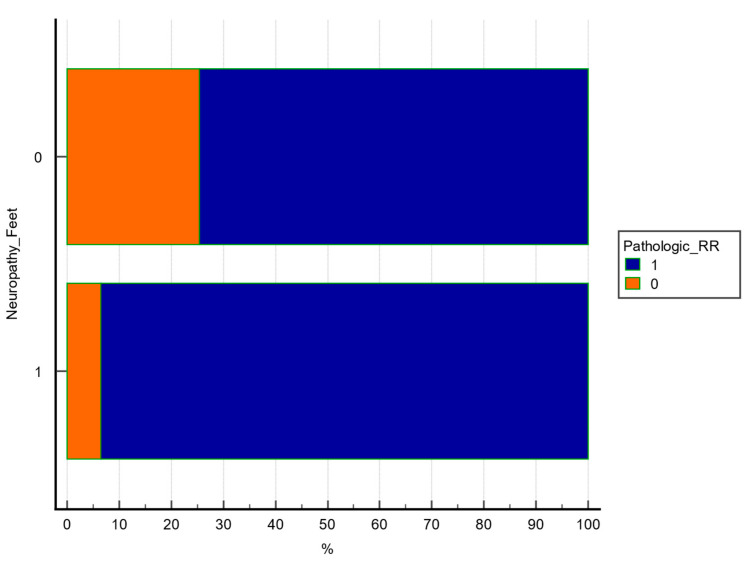
Distribution of pathological RR interval by SUDO Feet+.

Chi square test was used to explore the association between 2 categorical variables. A statistically significant association was observed between lower extremity sudomotor dysfunction and pathologic RR ratio (χ^2^ = 6.521, df = 1, *p* = 0.0107, contingency coefficient = 0.238), as shown in Table 5 and Figure 1.

The figure illustrates the percentage of patients with or without abnormal RR in each SUDO subgroup. A significant difference was observed *(p* = 0.0107, chi-square test).

**Figure 2:**
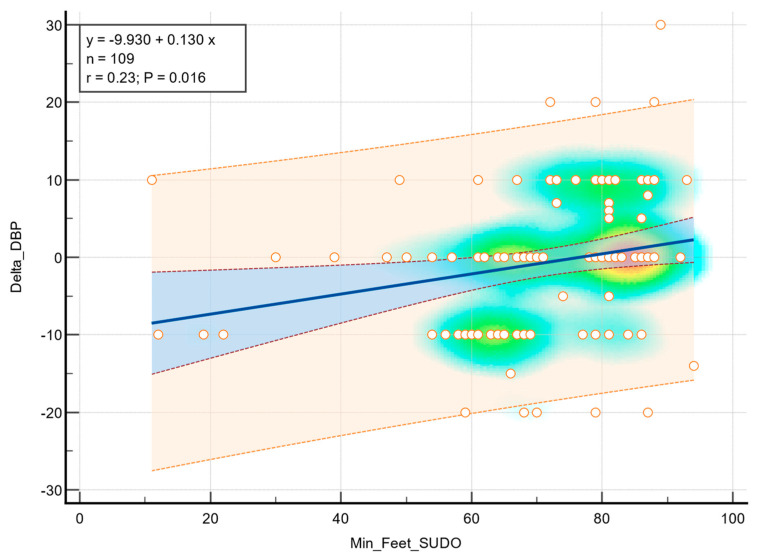
Correlation between feet ESC and diastolic blood pressure drop. The background color gradient represents the density of data points, with green and yellow areas indicating higher concentrations of values. Circles represent individual data points. The blue line indicates the linear regression, and the shaded area represent the 95% confidence interval.

**Figure 3:**
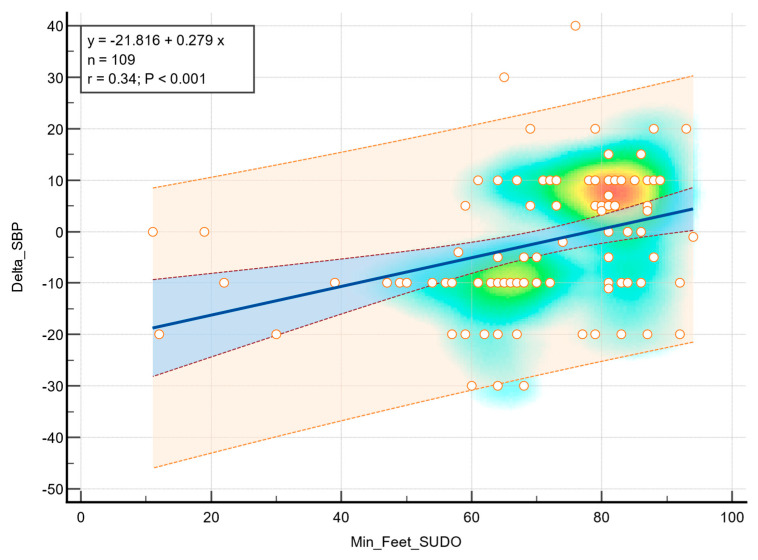
Correlation between feet ESC and systolic blood pressure drop. The background color gradient represents the density of data points, with green and yellow areas indicating higher concentrations of values. Circles represent individual data points. The blue line indicates the linear regression, and the shaded area represent the 95% confidence interval.

Linear regression analysis demonstrated a statistical association between feet ESC values and diastolic blood pressure during the orthostatic transition, as illustrated in Figure 2. Moderate correlation was observed (r = 0.23, *p* = 0.016), indicating that greater sudomotor dysfunction was associated with a higher decrease in diastolic blood pressure in response to standing. In addition, a statistically significant correlation was observed between the feet ESC values and a decrease in systolic blood pressure (r = 0.34, *p* < 0.001), as shown in Figure 3.

**Figure 4:**
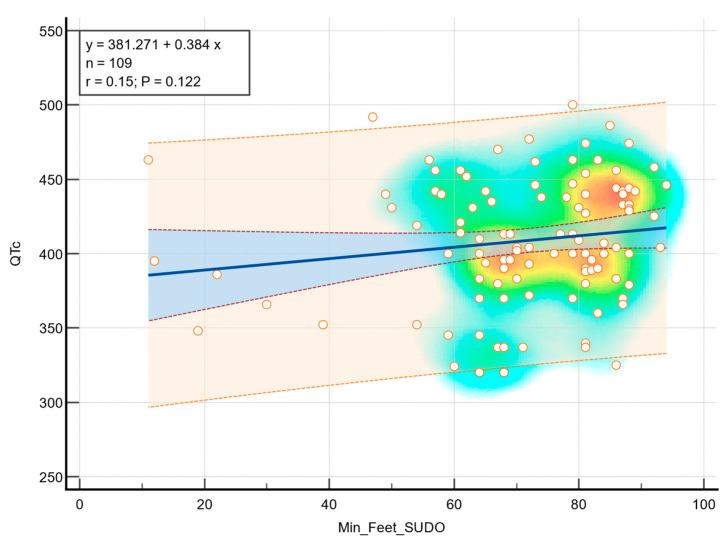
Correlation between foot ESC and QTc. The background color gradient represents the density of data points, with green and yellow areas indicating higher concentrations of values. Circles represent individual data points. The blue line indicates the linear regression, and the shaded area represent the 95% confidence interval.

Although a positive trend was observed in terms of foot ESC and QTc, there was no statistically significant correlation (r = 0.15, *p* = 0.122), as can be observed in Figure 4.

**Figure 5:**
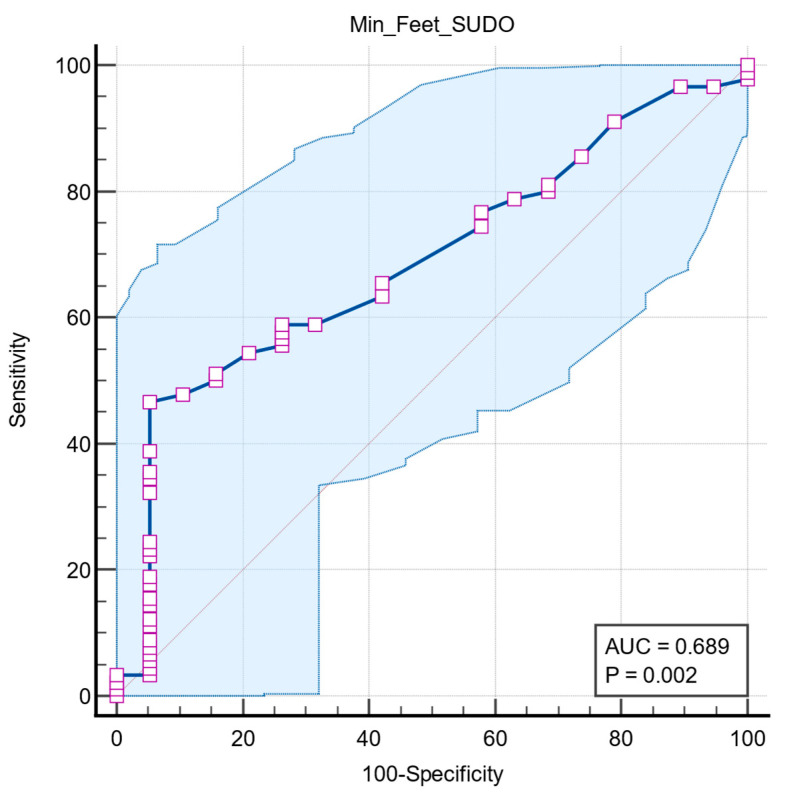
Graphical representation of ROC curve analysis of the SUDOSCAN in predicting a pathological RR ratio. The blue line represents the ROC curve, and the diagonal grey line indicates the line of no discriminations. The shaded area represents the 95% confident interval. Purple squares indicate the data points used to construct the ROC curve. AUC: area under the curve.

Receiver operating characteristic (ROC) analysis was used to assess the association of lower extremity ESC values with pathologic RR ratio. The optimal cutoff was determined by the maximum Youden index. ROC analysis showed that foot ESC has moderate power to identify autonomic dysfunction, with an AUC of 0.689 (95% CI: 0.593–0.774, *p* = 0.0022). A cutoff of ≤68 µS shows high specificity (94.7%) but moderate sensitivity (46.7%) (Figure 5).

**Figure 6:**
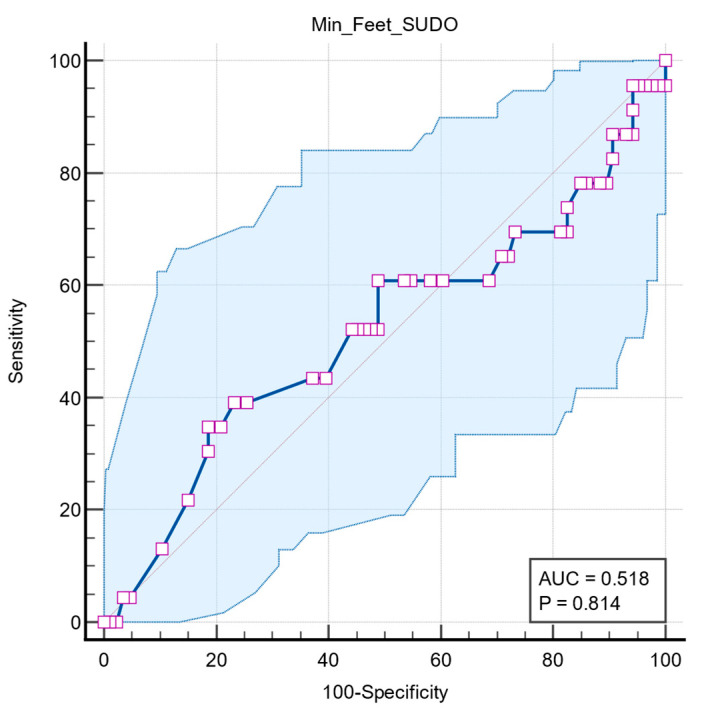
Graphical representation of ROC curve analysis of the SUDOSCAN in predicting long QT interval. The blue line represents the ROC curve, and the diagonal grey line indicates the line of no discriminations. The shaded area represents the 95% confident interval. Purple squares indicate the data points used to construct the ROC curve. AUC: area under the curve.

**Figure 7:**
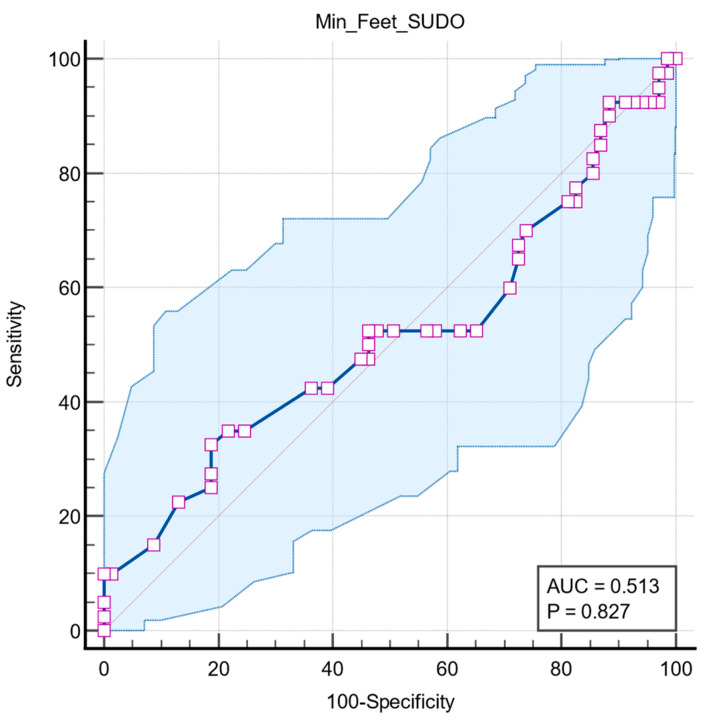
Graphical representation of ROC curve analysis of the SUDOSCAN in predicting orthostatic hypotension. The blue line represents the ROC curve, and the diagonal grey line indicates the line of no discriminations. The shaded area represents the 95% confident interval. Purple squares indicate the data points used to construct the ROC curve. AUC: area under the curve.

Two further ROC analyses were performed to determine whether the predictive value of feet ESC is associated with other autonomic dysfunctions, such as long QT interval and orthostatic hypotension. Both results had no statistical significance, with QTc interval having an AUC = 0.518 (95% CI: 0.420 to 0.615, *p* = 0.814) (Figure 6) and orthostatic hypotension having an AUC = 0.513 (95% CI: 0.416 to 0.610, *p* = 0.827) (Figure 7).

## 4. Discussion

### 4.1. Findings and Interpretation

CAN increases mortality rates among diabetics and is an independent risk factor for cardiovascular disorders. As T2DM patients can have a silent progression and a sizable percentage already have related chronic complications at the time of diagnosis, the American Diabetes Association (ADA) advises screening for neuropathy symptoms starting five years after type 1 diabetes mellitus (T1DM) diagnosis and at the time of T2DM diagnosis [11].

As a subset of autonomic function, sudomotor function shows the integrity of sympathetic nerves fibers that innervate the sweat glands. Sudomotor dysfunction in diabetics is typified by a reduced sweat response, which is especially apparent in the lower limbs. This study investigated the relationship between sudomotor dysfunction, quantified by measuring ESC values using SUDOSCAN, and the presence of CAN in patients diagnosed with T2DM.

In the present study, low ESC values of feet were significantly associated with decreased RR interval ratio, emphasizing that sudomotor dysfunction mirrors cardiac autonomic impairment. Additionally, patients with lower ESC values showed impaired systolic and diastolic blood pressure in response to standing, suggesting baroreflex dysfunction. Similar results were reported by Yajnik et al. [12] who found that patients with low ESC values showed longer duration of diabetes, elevated HbA1C values, renal function decline, and older age, except for renal function, to which ESC values were not related.

Peripheral neuropathy was assessed using tools such as TCS and MNCI. Similar results were found by Carbajal-Ramirez et al. [13], who also confirmed the correlation between lower ESC values and higher clinical neuropathy scores. The study compared ESC values measured in hands and feet with MNSI results and how effectively SUDOSCAN detects neuropathy. In all, 221 individuals were enrolled and separated into two groups: those who had been diagnosed with T2DM for less than five years and those who had been diagnosed for at least five years. The physical examination for MNSI involved assessing muscular stretch reflexes, skin abnormalities, vibration feeling with a 128 Hz tuning fork on both feet, and pressure sensitivity with a 10 g monofilament. Both the hands and the feet underwent the SUDOSCAN instrumental evaluation. Patients with neuropathy as determined by MNSI showed reduced conductivity in their hands and feet in both groups. When using MNSI as a reference, unusual ESC values in the hands or feet (less than 60 μS or 70 μS, respectively) showed a 97% sensitivity and an 87% positive predictive value for identifying neuropathy in patients with longer-term diabetes. The sensitivity of abnormal hands or feet ESC values for neuropathy identification in patients with diabetes for less than five years was 91%, and its positive predictive power was 88%.

Over the past years, a number of studies have assessed SUDOSCAN’s effectiveness and repeatability in DN detection. In their evaluation of various neuropathy tests on 265 diabetic patients, Yajnik et al. [12] discovered that the left and right sides’ ESC measurements differed by 9.5% for hands and 6.0% for feet, while the vibration perception threshold test showed a variation of 14.2%. Increased physical abnormalities on the MNSI B and increased symptoms on the MNSI A were both significantly correlated with lower values of ESC.

At an odds ratio of 4.41 (95% CI 1.72–11.29), individuals with an ESC of less than 40 μS were over four times as likely to have more than one abnormal cardiac autonomic neuropathy test than patients with an ESC of more than 40 μS. The fact that 40 μS is significantly less than what is considered the typical cutoff limit is noteworthy. Particularly, a postural drop in blood pressure, a sign of sympathetic cardiac autonomic neuropathy, was linked to lower ESC values. We used a threshold value of ≤68 μS of foot ESC in the present study. Krieger et al. [14] recommended 80 μS for people with type 2 diabetes, while Selvarajah et al. [15] recommended a cutoff threshold of 77 μS for subjects with type 1 diabetes (T1DM). Because ethnicity may actually have an impact on ESC, as Vinik et al. [16] described, it would make sense to utilize a cutoff value of foot ESC based on population ethnicity. According to Vinik et al. [16], ethnicity was not specifically assessed in our study and should be considered a limitation.

SUDOSCAN’s sensitivity, specificity, and repeatability were assessed in 133 T2DM patients by Mayaudon et al. [9] in comparison to 41 HVs. ESC displayed an area under the ROC curve of 0.88, a sensitivity of 75%, and a specificity of 100%. According to earlier research [17,18,19], our findings corroborate the well-established theory that distal areas, especially the feet, are more vulnerable to early neuropathic alterations because of the length-dependent degeneration of tiny fibers. This explains why measures from the upper extremities showed decreased performance, whereas feet ESC had the best diagnostic value.

D’Amato et al. [20] suggested a study to look at the ESC measure’s diagnostic performance for CAN and diabetic peripheral neuropathy (DPN), particularly when used in conjunction with a suitable questionnaire. After being diagnosed with CARTs, CAN was evaluated further using the COMPASS 31 questionnaire. In addition to the analysis of the vibration perception threshold (VPT), which was measured by a biothesiometer at the hallux dorsum and at the lateral malleolus, and the detection of the warm (WTT) and cold thermal perception thresholds (CTT), which were determined through the TSA-II Neurosensory Analyzer at the dorsum on both feet, DPN was evaluated using additional questionnaires (MNSI-Q and the Michigan Diabetic Neuropathy Score, MDNS). ESC measurements were deemed abnormal if they were less than 50 μS for the hands and less than 70 μS for the feet. The study included 102 individuals with diabetes who were at risk for developing diabetic foot syndrome. According to the most relevant results, the COMPASS 31 questionnaire and ESC had specificities of 67% and 65% for DPN and sensitivities of 83% and 75% for CAN, respectively. The specificity for DPN increased to 89% and the sensitivity for CAN to 100% when ESC and COMPASS 31 were combined. These results demonstrate the benefit of combining the effectiveness of two diagnostic methods of sudomotor measurement with validated instruments based on symptomatology.

The results of our study reinforce the fact that SUDOSCAN is an effective method for diagnosing CAN, especially when combined with other diagnostic methods.

### 4.2. Strengths and Limitations of the Study

One of the strengths of the study is the combination of cardiovascular autonomic tests and SUDOSCAN, a modern, non-invasive device, allowing multiple assessments of autonomic dysfunction. By integrating both diagnostic methods, the study provides statistical relevance for ESC values as a potential marker of CAN. Validated methods and tests were also used, which are accepted in the field and therefore increase the credibility of the results. The population consisted exclusively of patients with T2DM, being a relatively consistent group in disease etiology and treatment, and the population of the study presented heterogeneous characteristics.

Given the strengths, several limitations must be pronounced. The cross-sectional design of the study does not allow us to show a cause-effect relationship between sudomotor dysfunction and CAN. Second, the number of study participants is relatively small and comes from a single center, which may limit the applicability of the results to other populations. Another limitation is the absence of a healthy control group, which could help us to better find the clinical value of ESC. We also cannot know how the ESC value predicts neuropathy progression, as the study does not have a longitudinal design.

One limitation of our study is that we did not perform toe–brachial index (TBI) measurements. This may be particularly relevant in patients with diabetes, where arterial calcification can cause falsely elevated ABI values. Although ABI remains a useful screening tool, TBI could provide additional accuracy in this population [21].

This study was reported in accordance with the STROBE (Strengthening the Reporting of Observational Studies in Epidemiology) checklist for cross-sectional studies.

### 4.3. Relevance of the Findings

The result of the study contributes to support for the use of sudomotor testing as a practical marker for cardiac autonomic dysfunction in patients with diabetes. By showing that low ESC values are associated with pathologic RR variability and blood pressure in response to standing, the study indicates to us the potential of SUDOSCAN to detect certain changes that might go unseen in routine clinical assessments. Clinically speaking, the implementation of SUDOSCAN into diagnostic techniques may make it easier to identify high-risk patients early on who might benefit from more careful monitoring or prevention of diabetic neuropathy. The findings indicate SUDOSCAN’s use as a supplementary diagnostic technique rather than a stand-alone method to detect CAN in T2DM patients.

### 4.4. Future Perspectives

Multicenter and longitudinal investigations of larger and more varied populations of diabetic patients should be used in future study to validate these findings. The predictive utility of ESC in detecting early CAN and monitoring its development over time can be evaluated by this type of study. In addition, combining the use of SUDOSCAN with other tests for the identification of neuropathy integrated in diagnostic methods may improve clinical relevance. Given its simplicity, rapid application, and non-invasive nature, SUDOSCAN can be integrated into routine screening or diagnostic protocols in daily practice.

## 5. Conclusions

The present study demonstrated that low ESC values, as measured by SUDOSCAN, were associated with CAN in patients with T2DM, especially in those with pathologic RR ratio and abnormal blood pressure response to standing. Considering these results, SUDOSCAN can also be used as a complementary tool in the detection of CAN, especially when used in association with other standard diagnostic tests. Although its sensitivity invalidates it as the standalone screening method, its high specificity supports its potential for application in clinical practice.

## Figures and Tables

**Table 1:** Baseline characteristics of the study population.

Parameter	Result
Sex ^a^	Men: 56 (51.4%) Women: 53 (48.6%)
Age ^b^	63.6 years [56; 70]
Diabetes Duration ^b^	12 years [6; 20]
HbA1c ^b^	8.5% [7.3; 9.4]
BMI ^b^	31.3 kg/m^2^ [27.5; 34.6]
eGFR ^b^	75 mL/min/1.73 m^2^ [55; 88]
Sudomotor Neuropathy ^a^	SUDO+: 65 (59.6%) SUDO−: 44 (40.4%)
Orthostatic Hypotension ^a^	OH+: 40 (36.7%) OH−: 69 (63.3%)
Retinopathy ^a^	Retinopathy+: 35 (32.1%) Retinopathy−: 74 (67.9%)
PAD ^a^	PAD+: 21 (19.3%) PAD−: 88 (80.7%)
CV Events ^a^	CV+: 18 (16.5%) CV−: 91 (83.5%)
Insulin ^a^	Insulin+: 70 (64.2%) Insulin−: 39 (35.8%)
Metformin ^a^	Metformin+: 71 (65.1%) Metformin−: 38 (34.9%)

^a^ Continuous variables (with non-Gaussian distribution) are indicated by their median [interquartile range]. Mann–Whitney test. ^b^ Continuous variables (with Gaussian distribution) are indicated as mean ± standard deviation; HbA1c: hemoglobin A1c; BMI: body mass index; eGFR: estimated glomerular filtration rate; PAD: peripheral artery disease; CV: cardiovascular.

**Table 2:** Comparative analysis of sudomotor dysfunction.

	SUDO− *n* = 44	SUDO+ *n* = 65	*p*-Value *
Creatinine	0.98 0.90 to 1.06	1.17 1.03 to 1.31	0.3151
Delta_DBP	3.20 0.33 to 6.08	−3.26 −5.28 to −1.25	0.0001 **
Delta_SBP	4.23 0.46 to 7.99	−5.98 −9.18 to −2.79	0.0001 **
Diabetes Duration	10.45 8.26 to 12.65	14.43 12.42 to 16.44	0.0118 **
Min-Arm-SUDOSCAN	70.75 68.74 to 72.76	53.51 49.59 to 57.42	<0.0001
Min-Feet-SUDOSCAN	82.11 80.28 to 83.95	64.40 60.11 to 68.69	<0.0001
HbA1c	8.66 7.86 to 9.47	10.08 6.93 to 13.23	0.5226
Max-ABPI	1.11 1.07 to 1.15	1.09 1.05 to 1.14	0.5263
Min-ABPI	1.06 1.02 to 1.10	1.05 1.00 to 1.09	0.6207
BMI	31.17 29.42 to 32.91	31.61 30.45 to 32.78	0.7341
MNSI	4.75 4.03 to 5.47	6.60 6.10 to 7.10	<0.0001 **
Neutrophil lymphocyte ratio	2.02 1.80 to 2.24	3.46 1.22 to 5.70	0.9262
QTc	406.80 395.08 to 418.51	410.09 398.96 to 421.23	0.4754
RR ratio	1.09 1.05 to 1.12	1.04 1.00 to 1.07	0.0278 **
eGFR	79.93 73.10 to 86.77	65.64 59.89 to 71.40	0.0085 **
Toronto	4.50 3.98 to 5.02	6.11 5.67 to 6.55	<0.0001 **
Age	61.07 58.48 to 63.66	65.34 63.18 to 67.50	0.0098 **

* *p*-value was calculated using Mann–Whitney test; ** *p*-value < 0.05 (statistical significance). DBP: diastolic blood pressure; SBP: systolic blood pressure; HbA1c: hemoglobin A1c; ABPI: Ankle–brachial pressure index; BMI: body mass index; MNSI: Michigan Neuropathy Screening Instrument; QTc: corrected QT interval; eGFR: estimated glomerular filtration rate.

**Table 3:** Comparative analysis of sudomotor dysfunction on feet.

	SUDO Feet− *n* = 63	SUDO Feet+ *n* = 46	*p*-Value *
Creatinine	0.98 0.90 to 1.03	1.00 0.83 to 1.21	0.3798
Delta _DBP	0.00 0.00 to 5.00	−5.00 −10.00 to 0.00	<0.0001 **
Delta_SBP	5.00 1.00 to 10.00	−10.00 −10.00 to −10.00	<0.0001 **
Diabetes Duration	10.00 6.50 to 13.00	13.00 10.00 to 17.34	0.0214 **
Min-Arm-SUDOSCAN	66.00 62.25 to 70.00	59.50 50.66 to 65.00	0.0052
Min-Feet-SUDOCAN	81.00 81.00 to 84.75	63.50 59.00 to 64.11	<0.0001
HbA1c	8.37 7.80 to 8.80	8.50 8.19 to 8.92	0.9976
Max-ABPI	1.10 1.07 to 1.12	1.08 1.00 to 1.14	0.3442
Min-ABPI	1.03 1.00 to 1.08	1.03 0.98 to 1.10	0.5614
BMI	32.00 29.82 to 33.78	31.21 29.27 to 32.06	0.7801
Neutrophil lymphocyte Ratio	1.89 1.68 to 2.04	1.89 1.56 to 2.31	0.5095
QTc	413.00 402.50 to 436.75	396.00 383.00 to 414.56	0.0334 **
RR ratio	1.11 1.08 to 1.14	1.04 0.90 to 1.10	0.0013 **
eGFR	79.00 74.96 to 84.77	67.95 52.73 to 74.20	0.0059 **
Toronto	4.00 4.00 to 6.00	7.00 6.00 to 7.00	<0.0001 **
Age	60.00 56.00 to 65.00	69.00 65.89 to 70.11	0.0003 **

* *p*-value was calculated using Mann–Whitney test; ** *p*-value < 0.05 (statistical significance). DBP: diastolic blood pressure; SBP: systolic blood pressure; HbA1c: hemoglobin A1c; ABPI: Ankle–brachial pressure index; BMI: body mass index; MNSI: Michigan Neuropathy Screening Instrument; QTc: corrected QT interval; eGFR: estimated glomerular filtration rate.

**Table 4:** Comparative analysis of sudomotor dysfunction on arms.

	SUDO Arms− *n* = 67	SUDO Arms+ *n* = 42	*p*-Value *
Creatinine	0.97 0.89 to 1.01	1.07 0.85 to 1.28	0.1422
Diabetes Duration	12.00 9.00 to 15.00	12.00 10.00 to 15.00	0.4352
Delta_DBP	0.00 0.00 to 0.00	0.00 −4.08 to 0.00	0.2665
Delta_SBP	0.00 −5.00 to 5.00	−10.00 −10.00 to 3.26	0.1991
HbA1c	8.40 7.80 to 8.80	8.50 8.08 to 8.90	0.7603
Max-ABPI	1.10 1.07 to 1.14	1.07 1.00 to 1.13	0.2632
Min-ABPI	1.04 1.00 to 1.10	1.02 0.97 to 1.09	0.3560
BMI	31.21 29.39 to 32.32	31.73 29.47 to 33.95	0.4895
MNSI	6.00 5.00 to 7.00	7.00 7.00 to 7.00	0.0224
Neutrophil–lymphocyte ratio	1.98 1.76 to 2.21	1.70 1.51 to 1.93	0.4023
QTc	404.00 396.03 to 413.99	431.00 395.18 to 440.00	0.1805
RR ratio	1.08 1.04 to 1.12	1.10 1.05 to 1.12	0.8274
eGFR	77.36 72.81 to 83.47	69.93 53.37 to 77.00	0.0672
Toronto	6.00 4.00 to 6.00	6.00 6.00 to 7.00	0.0215
Age	64.00 59.01 to 67.00	66.50 61.37 to 70.00	0.3185

* *p*-value was calculated using Mann–Whitney test; * *p*-value < 0.05 (statistical significance). DBP: diastolic blood pressure; SBP: systolic blood pressure; HbA1c: hemoglobin A1c; ABPI: Ankle–brachial pressure index; BMI: body mass index; MNSI: Michigan Neuropathy Screening Instrument; QTc: corrected QT interval; eGFR: estimated glomerular filtration rate.

**Table 5:** Association between pathological RR ratio and sudomotor dysfunction (SUDO Feet+).

	Neuropathy_Feet		
**Pathologic_RR**	**0**	**1**	
0	16 84.2% RT 25.4% CT 14.7% GT	3 15.8% RT 6.5% CT 2.8% GT	19 (17.4%)
1	47 52.2% RT 74.6% CT 43.1% GT	43 47.8% RT 93.5% CT 39.4% GT	90 (82.6%)
	63 (57.8%)	46 (42.2%)	109
**Chi-squared**	6.521		
DF	1		
Significance level	*p* = 0.0107		
Contingency coefficient	0.238		

Chi-square test was used to assess statistical significance. Frequencies are presented as number of patients and percentages. RT: percentage from row total; CT: percentage from column total; GT: percentage from global.

## Data Availability

The data presented in this study are available on request from the corresponding author due to restrictions imposed by the General Data Protection Regulation procedure in the hospitals in which this study was conducted.
